# Investigation of the Potential Mechanism Governing the Effect of the *Shen Zhu San* on COVID-19 by Network Pharmacology

**DOI:** 10.1155/2020/8468303

**Published:** 2020-11-07

**Authors:** Yuxuan Wang, Yuhua Ru, Guowei Zhuo, Maozheng Sheng, Shuangqiu Wang, Jiarui Ma, Chongyi Zhou, Xiaohe Sun, Yanqi Zeng, Ya Zhang, Hui Li, Zhigang Lu, Depei Wu, Minghua Wu

**Affiliations:** ^1^Department of Neurology, Affiliated Hospital of Nanjing University of Chinese Medicine, Jiangsu Province Hospital of Chinese Medicine, Nanjing 210029, Jiangsu, China; ^2^Key Laboratory of Acupuncture and Medicine Research of Ministry of Education, Nanjing University of Chinese Medicine, Nanjing 210046, China; ^3^State Key Laboratory Cultivation Base for TCM Quality and Efficacy, Nanjing University of Chinese Medicine, Nanjing 210046, Jiangsu, China; ^4^National Clinical Research Center for Hematologic Diseases, Jiangsu Institute of Hematology, The First Affiliated Hospital of Soochow University, Suzhou 215006, Jiangsu, China; ^5^First Clinical Medical School, Affiliated Hospital of Nanjing University of Chinese Medicine, Jiangsu Province Hospital of Chinese Medicine, Nanjing 210046, Jiangsu, China; ^6^Provincial Key Laboratory of Drug Target and Drug for Degenerative Disease, School of Medicine and Holistic Integrative Medicine, Nanjing University of Chinese Medicine, Nanjing 210046, Jiangsu, China; ^7^Department of Respiration, Affiliated Hospital of Nanjing University of Chinese Medicine, Jiangsu Province Hospital of Chinese Medicine, Nanjing 210029, Jiangsu, China

## Abstract

**Background:**

Since December 2019, coronavirus disease 2019 (COVID-19) due to SARS-CoV-2 infection has emerged in Wuhan and rapidly spread throughout China and even to other countries. Combined therapy with modern medicine and traditional Chinese medicine has been proposed, in which *Shen Zhu San* (SZS) was regarded as one of the basic prescriptions.

**Methods:**

Network pharmacological approaches along with candidate compound screening, target prediction, target tissue location, protein-protein interaction network, gene ontology (GO), KEGG enrichment analyses, and gene microarray analyses were applied.

**Results:**

A total of 627 targets of the 116 active ingredients of SZS were identified. Targets in immune cells and tissues were much more abundant than those in other tissues. A total of 597 targets were enriched in the GO biological cellular process, while 153 signaling pathways were enriched according to the KEGG analysis. A total of 450 SARS-related targets were integrated and intersected with the targets of SZS to identify 40 common targets that were significantly enriched in five immune function aspects of the immune system process during GO analysis. Several inflammation-related pathways were found to be significantly enriched throughout the study.

**Conclusions:**

The therapeutic mechanisms of the effects of SZS on COVID-19 potentially involve four effects: suppressing cytokine storms, protecting the pulmonary alveolar-capillary barrier, regulating the immune response, and mediating cell death and survival.

## 1. Introduction

In early December 2019, a series of pneumonia cases of unknown cause were first confirmed in Wuhan, Hubei Province, China [1]. According to the World Health Organization (WHO), by June 30, 2020, there have been over ten million cases and five hundred thousand deaths in 215 countries and regions worldwide. Unfortunately, the number is still increasing. Efforts made by the Chinese Government to curb outbreaks included initiating first measures to isolate Wuhan, which were extended to the whole Hubei province, standing 35 million residents during the Chinese Spring Festival [2]. Meanwhile, 30 provinces of China had launched a first-level response to the major public health emergencies [3]. Because of the severity of this outbreak, the WHO declared it a Public Health Emergency of International Concern on January 30, 2020 [4], and then increased assessment of the risk of spread and impact of COVID-19 to very high at a global level on February 28, 2020 [5].

Chinese scientists rapidly isolated and confirmed a novel type of coronavirus named SARS-CoV-2 by the International Committee on the Taxonomy of Viruses on February 11, 2020 [6, 7]. Coronaviruses are positive-sense single-stranded RNA viruses, which belong to the family Coronaviridae. SARS-CoV-2 is the seventh member of the coronavirus family and the third coronavirus that causes human fatal illness after SARS-CoV and MERS-CoV [8]. SARS-CoV, which caused outbreaks of severe acute respiratory syndrome (SARS) in Guangdong Province in China in 2002, shares 79.5% genetic sequence similarity with SARS-CoV-2 as well as the same cell entry receptor [8], and the amino acid sequence identity between the SARS-CoV-2 and the SARS-CoV S-proteins is 76.47% [9]. Some preliminary studies suggested that SARS-CoV-2 was similar to SARS-CoV to some extent, based on full-length genome phylogenetic analyses [8, 10] and the putative similarity of cell entry mechanism and human cell receptor utilization [8, 11, 12]. The disease caused by the novel coronavirus was named coronavirus disease 2019 (COVID-19) by the WHO [7]. The clinical signs and symptoms have been reported mainly as viral pneumonia with fever and dyspnea, a few even developed acute respiratory distress syndrome (ARDS), with chest radiographs showing bilateral pulmonary infiltration [1, 13–15].

As there are no specific antiviral therapies for COVID-19 and the main treatments are supportive at present, the Proposed Diagnosis and Treatment (*5*^*th*^*edition*) issued by China's National Health Commission integrated therapy with traditional Chinese medicine (TCM) and modern medicine [16]. To date, TCM intervention has been officially specified as an integrant therapeutic strategy in several provinces in China for patients who develop COVID-19 symptoms. The experts in TCM who were assigned by China's National Health Commission supplied a series of therapies to treat the COVID-19. Among these therapies, a traditional formula called *Shen Zhu San* (SZS, in Chinese: **神术散**) was regarded as one of the basic prescriptions for the treatment of COVID-19 [17–19].

SZS, which is a thousand-year-old TCM formula, was first recorded in an ancient Chinese medical book called “Yang's Hereditary Medical Formulary” and was later included in the “Prescriptions of Peaceful Benevolent Dispensary”, the official medical book of the Song Dynasty. SZS is mainly composed of seven medicinal herbs: *Atractylodis Rhizoma*, *Angelicae Dahuricae Radix*, *Asari Radix Et Rhizoma*, *Glycyrrhizae Radix Et Rhizoma*, *Notopterygii Rhizoma Et Radix*, *Ligustici Rhizoma Et Radix*, and *Chuanxiong Rhizoma* [20, 21]. In the long course of history, SZS has been widely used throughout China and has been proven to have therapeutic effects on epidemic infectious diseases. Modern toxicological studies demonstrated that the herbs in SZS have a high margin of drug safety [22–29]. According to ancient records and modern application, SZS is good for treating respiratory tract diseases with fever, shortness of breath, cough, nasal obstruction, headache, dizziness, body aches, and hematochezia caused by the wind-cold damp pathogen, for which the symptoms and TCM pathogenesis are highly consistent with those COVID-19. As a new pharmacological discipline, integrating network pharmacology emphasizes the concept of a “multicomponent, multitarget therapeutic network” and highlights the overall concept of TCM [30]. Network pharmacology offers a novel concept for understanding the multitargeted mechanism of the treatment of complex diseases with TCM [31]. In this study, we explored the potential mechanism of SZS in COVID-19 treatment by utilizing integrating network pharmacological approaches.

## 2. Results

### 2.1. Potential Mechanism of SZS in COVID-19 Treatment

#### 2.1.1. Identification of Active Compounds and Targets of SZS

Using the TCMSP and SymMap databases, the chemical ingredients were identified based on the criteria of OB ≥ 30% and DL ≥ 0.18: 9 in *Atractylodis Rhizoma*, 18 in *Angelicae Dahuricae Radix*, 6 in *Asari Radix Et Rhizoma*, 71 in *Glycyrrhizae Radix Et Rhizoma*, 12 in *Notopterygii Rhizoma Et Radix*, 1 in *Ligustici Rhizoma Et Radix*, and 7 in *Chuanxiong Rhizoma*. There were 116 chemical ingredients chosen for further investigation after the removal of duplicates. A total of 2219 human component-target interactions were generated from the 116 active ingredients of SZS. Duplicates of the validated and predicted component targets were eliminated, and then the 627 targets of the active ingredients of SZS were screened (Table S1), among which the main compounds and targets are shown in Table 1.

#### 2.1.2. Compound-Target Network and Target Tissue Location

**Figure 1:**
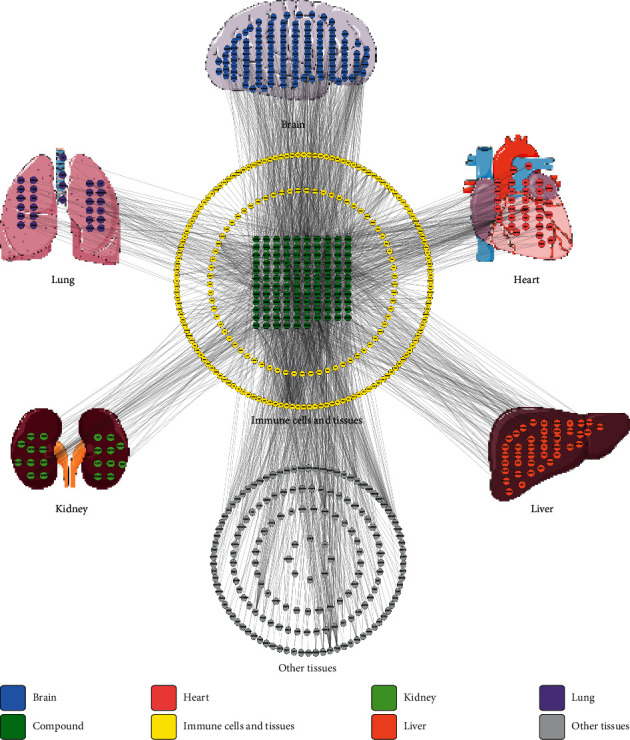
Compound-target-organ location map. The displayed nodes collectively represent the organs in which each target was located. The nodes with different colors represent the corresponding relationships of targets and organizations.

The 627 targets of the active ingredients of SZS mentioned above were used to construct a component-target network classified by tissue. Genes are commonly expressed in multiple tissues and cell types. In this study, as shown in Figure 1, the targets were located in certain tissues where they had the highest mRNA expression level according to the BioGPS database [32, 33], because we were interested in the expression patterns of the targets in specialized cells and tissues, including immune cells and tissues, lung, brain, heart, kidney, and liver. The mRNA-specific expression patterns of targets offer significant clues for understanding the underlying therapeutic effects of SZS. Specifically, there were 207 targets located in immune cells and tissues: 28 in the lung, 126 in the brain, 34 in the heart, 20 in the kidney, and 42 in the liver. The nodes with different colors represent the corresponding relationships of targets and organizations, as illustrated in the lower-left corner of Figure 1. The number and the total degree of all the nodes of component targets in immune cells and tissues (marked in yellow) were much greater than those in other tissues, which indicated that SZS might exert therapeutic effect by regulating immune function.

#### 2.1.3. Analysis of Targets in PPI Network

**Figure 2:**
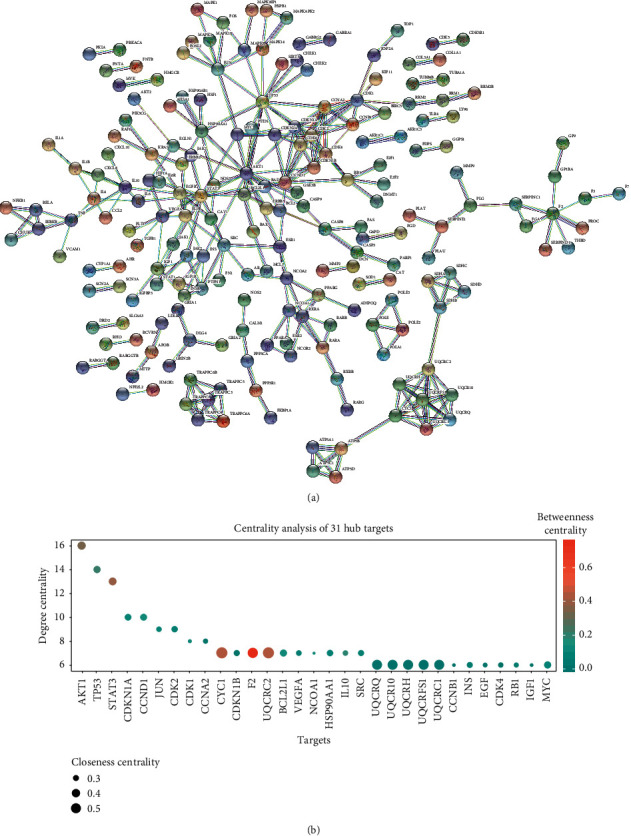
The PPI network for SZS and hub target analysis. (a) The nodes indicate proteins, and the edges represent protein-protein associations. The cyan edges represent interactions from the curated database, and the purple edges were experimentally determined. The green, red, and blue edges represent the interactions that were determined according to the gene neighborhood, fusions, and co-occurrence, respectively. (b) The centrality of targets was evaluated according to the degree centrality, betweenness centrality, and closeness centrality, which exhibited variation in terms of the *y*-axis values, colors of nodes, and sizes of nodes.

A PPI network was constructed to analyze and understand the mechanisms of the effects of SZS according to the protein-protein interactions. A total of 627 targets were inputted into the STRING database and the target proteins were rejected independently of the PPI network (Figure 2(a)) [34]. There were 201 nodes (representing active proteins) and 292 edges (representing the interaction between the active proteins and other proteins) in the network. All the nodes represented queried proteins and the first layer of interactors, and the protein-protein associations represented as edges were implied to be specific and meaningful. The results of the network topology analyses were as follows: the average degree of the nodes was 2.9054, and there were 81 nodes with a degree higher than the average degree. The average betweenness centrality of nodes was 0.0562, and there were 34 nodes with a higher than average betweenness centrality. The significance of key proteins was analyzed based on the comprehensive assessment of the degree, betweenness centrality, and closeness centrality of the nodes exported from the STRING database. According to a threshold degree value of ≥6, 31 critical nodes were further identified and are shown in bubble charts (Figure 2(b)).

#### 2.1.4. GO Functional and KEGG Pathway Enrichment Analysis

**Figure 3:**
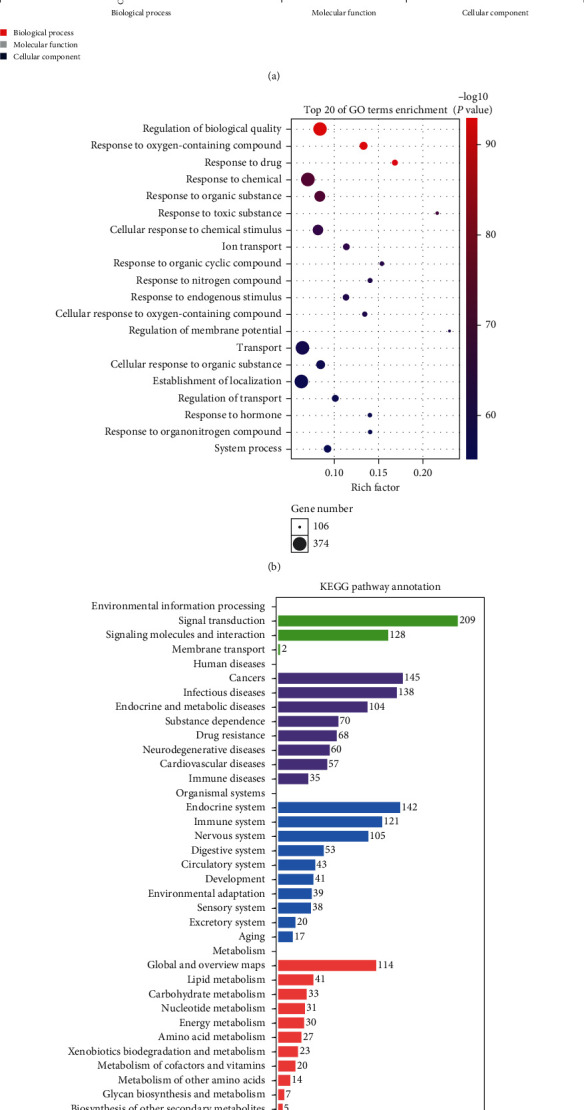
GO functional and KEGG enrichment analysis. (a) The second GO class enrichment statistics for 627 targets. The *x*-axis represents the GO terms: red for BP terms, gray for MF terms, and blue for CC terms. The *y*-axis shows the number of enriched genes. (b) The bubble diagram shows the top 20 BP terms on the *y*-axis, while the annotated gene counts for the BP terms are presented on the *x*-axis. (c) KEGG pathway annotation. The *x*-axis represents the number of genes in the given classification. The details of the GO and KEGG analyses are presented in Tables S2 and S3, respectively.

As a bioinformatics analysis tool, GO defines the input genes by describing their function and the relationships between concepts [35]. After collection of data by ClueGO and CluePedia, GO annotation and enrichment of the SZS target protein genes were performed for three concepts of cell composition, molecular function, and biological process. The GO enrichment analysis of 627 potential targets (Figure 3(a)) showed that the main BP terms enriched in the second GO class were cellular process, biological regulation, and response to the stimulus. For further exploration of the changes in these biological functions, we selected the top 20 BP in the third GO class to generate bubble charts according to the *p* value (Figure 3(b)). The results showed that the mechanism of SZS was mainly related to the regulation of biological quality, oxygen-containing compound response, drug response, chemical response, organic substance response, toxic substance response, chemical stimulus cellular response, ion transport, organic cyclic compound response, and nitrogen compound response. Among these processes, 597 genes were enriched in the BP involved in the cellular process, 531 genes were enriched in processes of biological regulation, and 515 genes were enriched in stimulus response.

There were a total of 153 significant signaling pathways identified by the KEGG enrichment analysis. The bar plot of the KEGG pathway annotation is presented in Figure 3(c). Environmental information processing was mainly enriched in terms of signal transduction and signaling molecules and interactions. Human diseases mainly involved cancers, infectious diseases, and endocrine and metabolic diseases. The data indicated that the targets of SZS were widely involved in regulating metabolism and cell growth and death. Furthermore, there were a few targets involved in genetic information processing. The specific pathways are illustrated in the Discussion section in detail.

### 2.2. Understanding SZS by Analogy: Investigation of the Potential Mechanism of the Effects of SZS in SARS-CoV Pneumonia Treatment

#### 2.2.1. Screening of SARS-Related Targets and Determination of Common Targets between SZS and SARS

To date, there have been few data available describing the molecular mechanisms underlying COVID-19 so far. To better understand the potential therapeutic effect of SZS on COVID-19, we investigated the potential mechanism of the effects of SZS in SARS-CoV pneumonia treatment. Eighteen years ago, there was an outbreak of SARS-CoV pneumonia in Guangdong, China. As one of the few lethal coronaviruses, SARS-CoV-2 shares 79.5% of its sequence with SARS-CoV and has a similar receptor-binding domain structure to that of SARS-CoV [8, 10], which is the reason that the International Committee on the Taxonomy of Viruses recognized the novel coronavirus as closely related to SARS-CoV. The International Committee on the Taxonomy of Viruses decided to formally name the novel coronavirus SARS-CoV-2 [6]. Regardless of the unclear pathogenesis, the novel coronavirus has caused clusters of fatal pneumonia with a clinical presentation greatly resembling SARS, including the development of ARDS and cytokine storm-caused lethality [1, 13, 14, 36, 37]. Moreover, front-line medical workers announced that the abnormalities in laboratory results and possible pathophysiology of COVID-19, such as cellular immune deficiency, coagulation activation, and multiple organ injury, were similar to those of SARS-CoV pneumonia [14, 15, 36]. Thus, investigating the underlying mechanisms of the effects of SZS in relieving SARS-CoV pneumonia may partly benefit the current fight against COVID-19.

**Figure 4:**
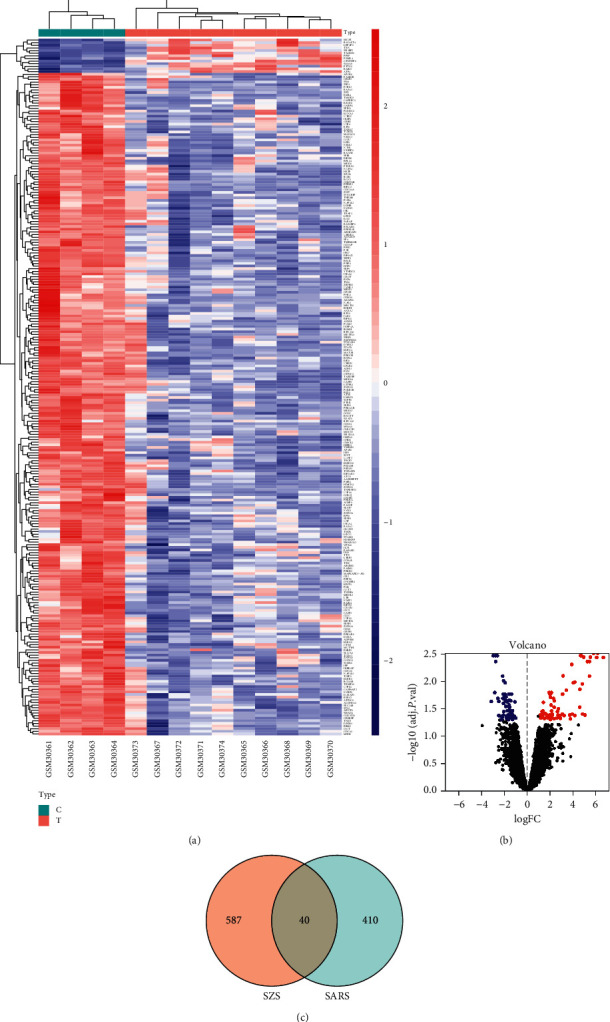
Differential gene expression heatmap and volcano plot for SARS-CoV and common targets between SZS and SARS. (a) Differential gene expression heatmap for SARS-CoV (all upregulated and downregulated genes). (b) Differentially expressed genes were selected by volcano plot filtering (fold change ≥ 1 and *p* value ≤ 0.05). (c) Venn diagram of the overlapping targets of SZS and SARS.

Hence, we integrated totally 450 disease targets related to SARS from the MalaCards database and GEO gene microarray analysis as described in Methods 5.7. The differentially expressed genes are presented in a heatmap (Figure 4(a)) and a volcano plot (Figure 4(b)). As illustrated by the Venn diagram (Figure 4(c)), the disease targets and the component targets intersected to identify 40 common targets, which implies the importance of understanding the role of SZS in COVID-19 treatment by analogy to that in SARS-CoV pneumonia treatment.

**Figure 5:**
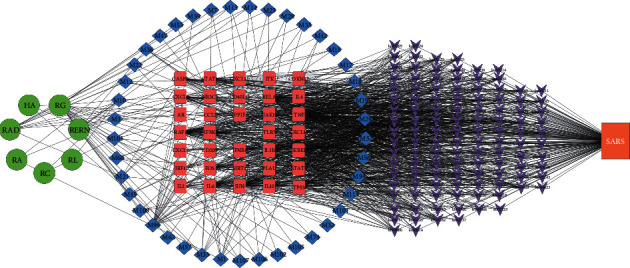
Herb-compound-target-pathway network. Nodes in different colors represent different groups: green for herbs, blue for compounds, red for cotargets, purple for pathways, and brown for the disease.

In addition, the compound-target networks as well as the relationships among the herb-compound-target-pathway interactions were constructed to better comprehend the complicated molecular mechanisms (Figure 5).

#### 2.2.2. Analysis of Cotargets in PPI Network

**Figure 6:**
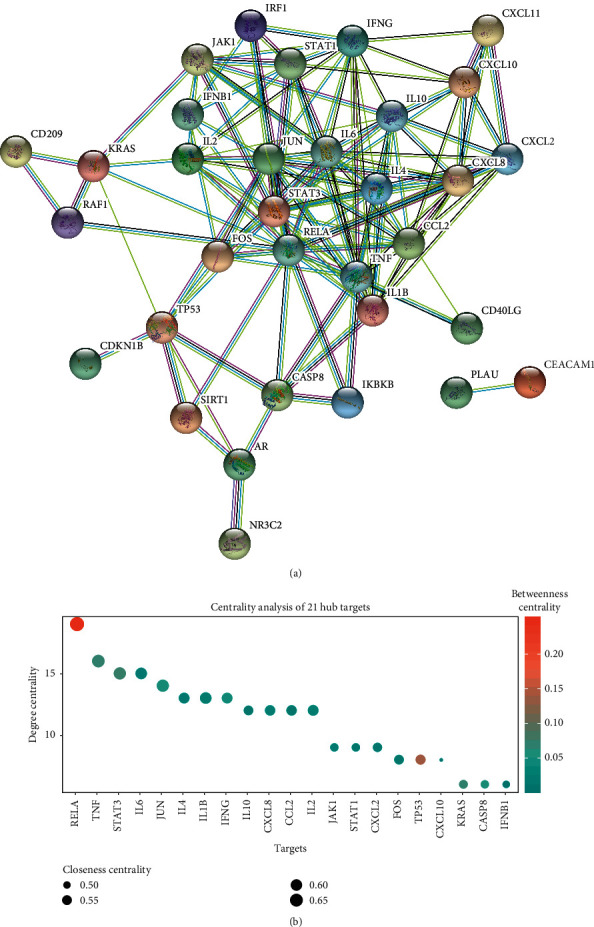
The PPI network for SZS and hub target analysis. (a) Network nodes indicate proteins, and edges represent protein-protein associations. (b) The centrality of targets was evaluated according to the degree centrality, betweenness centrality, and closeness centrality.

Forty cotargets were inputted into the STRING database according to the criteria of a confidence level greater than 0.9 and the rejection of target proteins excluded by the PPI network (Figure 6(a)). There were 33 nodes and 133 edges in the network. The results of the network topology analyses were as follows: the average degree of the nodes was 8.0606, and there were 15 nodes with an average degree higher than average. The average betweenness centrality of the nodes was 0.0324, and there were 9 nodes with higher than average betweenness centrality. The significance of the critical proteins was analyzed based on the degree, betweenness centrality, and closeness centrality of the node exported from STRING database. According to the criterion of a degree ≥6, 21 critical nodes were identified and are presented in bubble charts (Figure 6(b)).

#### 2.2.3. GO Functional Enrichment Analysis

**Figure 7:**
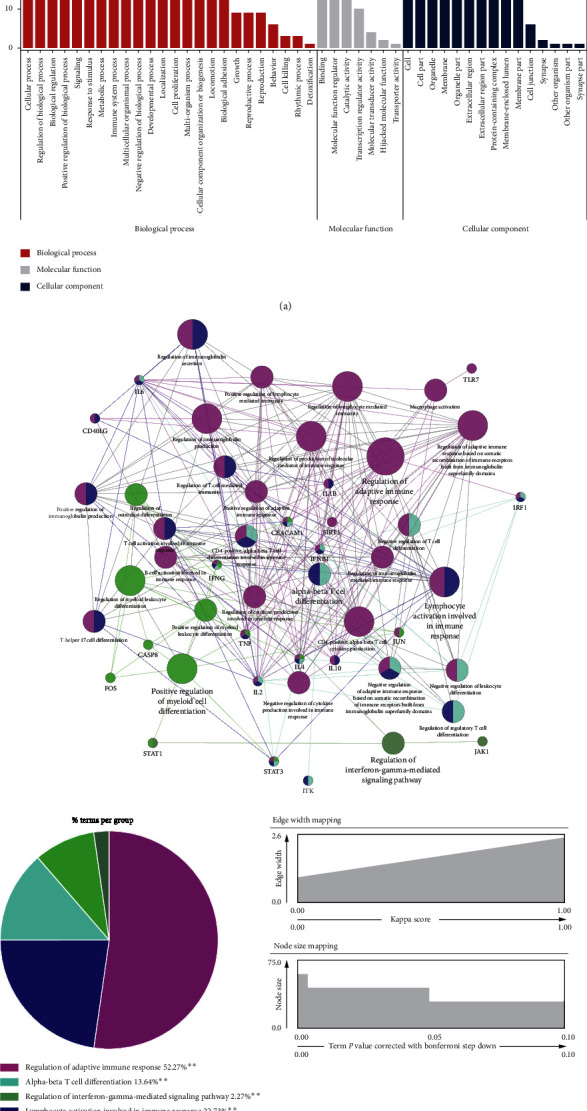
GO functional enrichment analysis and pathway mapping. (a) The second level GO enrichment statistics for 40 cotargets. (b) The cotarget genes were mapped for the immune system process GO terms by utilizing Cytoscape equipped with the ClueGO and CluePedia plugins. Each node represented a GO term, and its size represented the significance. An edge indicates the existence of common genes: a finer line indicates a smaller overlap. The different functional groups of GO terms were reflected by different node colors and are shown on the pie chart. ^*∗∗*^*p* < 0.01. The detailed statistics are provided in Table S4.

The most significantly enriched GO terms of the 40 cotargets are shown according to BP (red bar), MF (gray bar), and CC (blue bar) (Figure 7(a)). The main BP terms were cellular process, regulation of biological process, and biological regulation. Furthermore, the 40 cotargets were found to be enriched in the immune system process by utilizing the ClueGO and CluePedia plugins in Cytoscape (Figure 7(b)) [38–40]. The enrichment of the immune system process was determined to a kappa score of ≥0.4 and a *p* value set ≤0.01. The enriched GO terms were represented as nodes, whose sizes represented the significance of the enrichment of the term. The pie chart shows the proportion of each group associated with the 40 target genes (Figure 7(b)). There were 57 terms and 322 edges contained in the GO enrichment map. The terms were significantly enriched in terms of five immune functions: the regulation of the adaptive immune response, lymphocyte activation involved in the immune response, alpha-beta T-cell differentiation, positive regulation of myeloid cell differentiation, and regulation of the interferon-gamma-mediated signaling pathway.

#### 2.2.4. KEGG Pathway Enrichment Analysis

**Figure 8:**
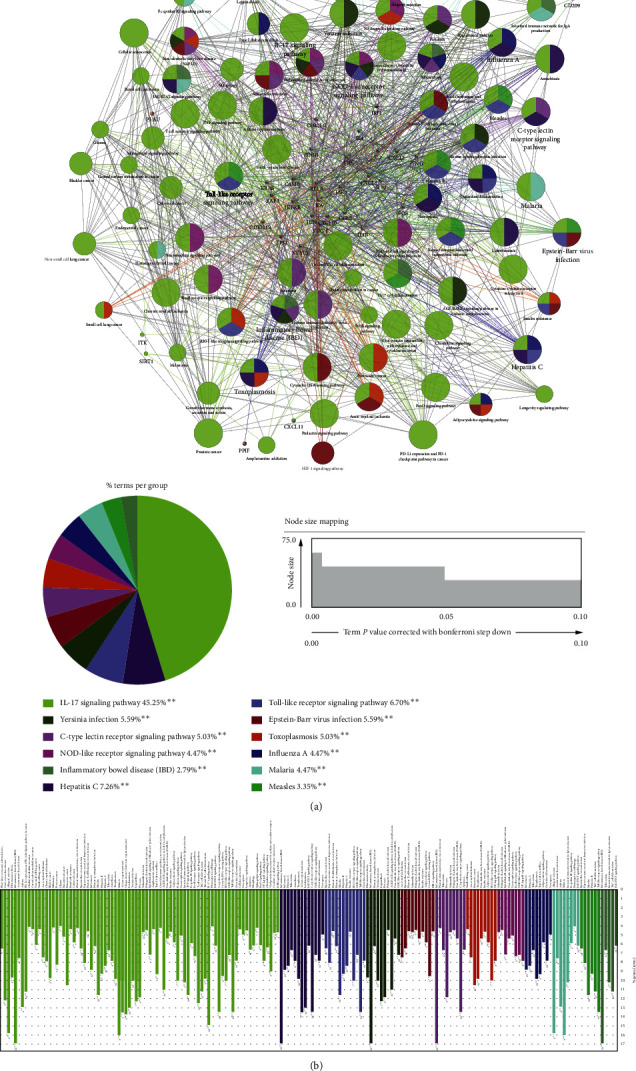
KEGG enrichment analysis and pathway mapping. (a) Functionally grouped network of the enriched categories was generated for the target genes using ClueGO and CluePedia plugins. Pathway terms were represented as nodes, and the node size represents the significance of the enrichment of the term. The pie chart shows the proportion of each group associated with 40 cotargets. (b) The bars show the number of genes related to the pathway terms. ^*∗∗*^*p* < 0.01 . The detailed statistics are provided in Table S5.

KEGG pathway analysis was conducted for the purpose of predicting the potential functions of the 40 cotargets. According to a kappa score of ≥0.4, 166 terms were found to be connected by 1247 edges (Figure 8(a)). The most significant KEGG terms for the target genes included the IL-17 signaling pathway, hepatitis C, Toll-like receptor signaling pathway, *Yersinia* infection, Epstein-Barr virus infection, C-type lectin receptor signaling pathway, toxoplasmosis, NOD-like receptor signaling pathway, influenza A, malaria, Measles, and inflammatory bowel disease. Beyond that, we noticed that several inflammation-related terms were also significantly enriched, such as the JAK-STAT signaling pathway, Th17 cell differentiation, NF-kappa B signaling pathway, RIG-I-like receptor signaling pathway, and HIF-1 signaling pathway (Figure 8(b)). These results were in accordance with those of the KEGG enrichment analysis for all the targets of SZS, suggesting that interventions in the inflammatory process might be the crucial mechanism involved in the treatment by SZS of the 2019 novel coronavirus or SARS-CoV pneumonia.

## 3. Discussion

### 3.1. SZS Is a Traditional TCM Formula Which Has Been Adopted to Treat COVID-19 in China

The manifestations of COVID-19 mimic those of SARS-CoV in terms of symptomatology, laboratory features, and chest imaging manifestations [1, 13–15], while there were still characteristics useful for differential diagnosis [14, 15, 36]. The most distinct symptoms of hospitalized COVID-19 patients were subsequent respiratory insufficiency caused by viral pneumonia, even followed by ARDS or acute respiratory failure [1, 13–15]. Combined with life support, TCM treatment has effectively reduced the severity and enhance the recovery of the COVID-19 in China [17, 18]. TCM already showed therapeutic effects on SARS during 2002–2003 [41–46]. Several TCM prescriptions have already been formulated for COVID-19 treatment, such as Ma *Xing Shi Gan Decoction*, *Qing-Fei-Pai-Du-Tang*, Lian Hua Qing Wen Capsule, and Shuang Huang Lian [47, 48], among which a classic formula named *Shen Zhu San* was regarded as one of the basic prescriptions [17–19, 49]. It is worth noting that different TCM prescriptions are suitable for COVID-19 cases with different symptoms. For example, Chinese doctors applied Ma *Xing Shi Gan Decoction* to treat COVID-19 patients who have a high-grade fever, while SZS is suitable for COVID-19 cases with a slight fever and body aches. SZS contains seven herbs that are made into a decoction that has been used to treat epidemic infectious diseases since the Song Dynasty in China. Some of the herbs and the compounds in SZS have been proven to have antiviral or anti-inflammatory influences *in vitro* or *in vivo* [50–55]. The database-based approaches are effective and seem to become prevalent in investigating the mechanism of TCM in treating COVID-19 [56, 57]. Our previous research utilized such approaches to systematically reveal the material basis and underlying biological mechanisms of *Ma Xing Shi Gan Decoction* in the treatment of COVID-19 [49]. In the present study, we utilized integrating network pharmacological approaches to investigate the potential mechanism of SZS involved in treating COVID-19.

The clinical manifestations, radiological evidence, and laboratory result abnormalities all hinted that the pulmonary pathological features were closely related to the inability of the lung to exchange gas, which is due to viral infection and persistent immune system responses, consequently resulting in downstream airway obstruction and alveolar structure loss [14, 15, 36, 58, 59]. Targeting the immune pathways that promote the inflammatory signals and contribute to diffuse alveolar injury may help to maintain vitality and earn a valuable time window for clearing the virus. Simultaneously, TCM therapy, such as SZS, could serve as an important adjuvant therapy [58, 60, 61]. Our data indicated that immune cells and tissues were the main targets of SZS (Figure 1), and the biological effect of SZS was widely involved in the regulation of the immune process, as illustrated in Figure 7(b).

### 3.2. SZS May Relieve COVID-19 through Suppressing Cytokine Storm

**Figure 9:**
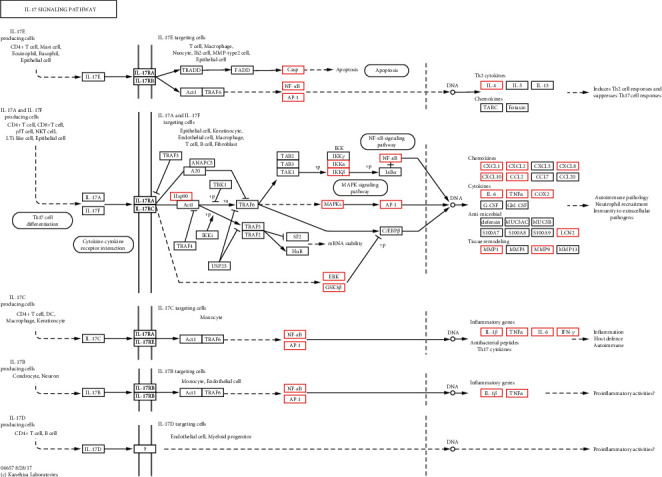
KEGG pathway: map04657. Red boxes mark the proteins or pathways targeted by SZS.

Resulting from virus-induced abnormal immune activation primarily, cytokine storm is an acute, lethal, and systemic complication connected with the severity of COVID-19 [1, 62]. Abnormal concentrations of several associated cytokines like TNF, IL-10, and IL-6 were found in COVID-19 patients [1, 63, 64]. As shown in Figure 9, SZS was likely to regulate inflammatory factor production through different mechanisms. Specifically, the TNF pathway was enriched in the KEGG enrichment analysis (Table S3), and TNF was a targeted protein in the PPI network (Figure 2(a)). TNF, a famous and perhaps the most intensely studied proinflammatory cytokine, is considered to play a prominent role in the cytokine storm and has been identified as a central cytokine in acute viral diseases, including those caused by the influenza virus, dengue virus, and Ebola virus [65, 66]. Besides, TNF is induced by secondary mediator cascades in respiratory epithelial cells after influenza virus infection, as opposed to a range of proinflammatory cytokines directly induced in respiratory epithelial cells [67, 68], which further indicates that TNF may act as a critical effector in amplifying and broadening the proinflammatory response and then escalating the cytokine storm [68, 69]. IL-10 is another core component of the cytokine system to prevent immunopathology during inflammatory responses [70]. Through the extracellular signal-regulated kinase/IL-10 axis, IL-10 is induced and stimulates STAT3, one of the 31 hub targets (Figure 2(b)), to inhibit the NF-*κ*B signaling pathway in the early phase of infection. Proinflammatory cytokines such as TNF are downregulated, and the cytokine storm can thereby be alleviated. IL-10 dysregulation tends to injure the host because of either excessive pathogen proliferation or cytokine storm [71]. Although the specific effects of IL-6 are mixed, IL-6 has a predominantly anti-inflammatory effect to protect the host from diverse infections and tissue injuries [72]. However, high levels of IL-6 may exert disproportionate effects like activating the coagulation pathway and vascular endothelial cells (ECs), thereby generating acute cytokine storms [62].

Several ingredients of SZS, such as quercetin, kaempferol, and wogonin, have been proven to reduce TNF and IL-6, thus suppressing inflammatory activity [65, 73–76]. What is more, quercetin could also regulate the cytokine balance by increasing serum IL-10 [77]. From these results and literature review, we came to the assumption that by decreasing TNF, quercetin, kaempferol, and wogonin could inhibit the dysfunction of ECs, extravasation of cytokines and inflammatory cells, and injury of the parenchymal cell. The ingredients in SZS may decrease tissue factor (TF) (Table S1) activated by excessive IL-6, thus decreasing the incidence of a lethal cytokine storm through suppressing the coagulation pathway. Besides, quercetin may also protect the host from cytokine storms by activating STAT3 to inhibit the NF-*κ*B/TNF-*α* axis. To be brief, quercetin, kaempferol, and wogonin in SZS potentially treat COVID-19 by suppressing cytokine storm.

### 3.3. Understanding the Role of SZS in COVID-19 by Analogy to SARS Treatment: SZS Could Participate in Regulating Immune Response and Mediating Cell Death and Survival

In the present study, due to the similarities between SARS-CoV pneumonia and COVID-19 from aspects of the etiology, clinical manifestation, and laboratory findings, we also attempted to understand SZS by analogy to the mechanisms of SZS in relieving SARS-CoV pneumonia. As illustrated in Figure 4, SZS shared 40 common targets associated with SARS. The acute phase of SARS is related to a severe reduction in the number of T cells in the blood [78].

Jun and Fos are contained in the 21 hub cotargets (Figure 6(b)) as well as the enriched GO terms of the immune system process (Figure 7(b)). c-Jun NH2-terminal kinases (JNKs) take part in cellular proliferation, differentiation, and death, as well as the response to proinflammatory molecules. In particular, they play a critical role in T helper cell (Th) activation and maintenance of Th1/Th2 polarization [79]. Fos regulates transcription of several cytokine genes affected by SARS-CoV infection, including the proinflammatory cytokines involved in the cytokine storm during SARS-CoV pathogenesis [80]. AP-1 consists of members of Jun and Fos families, and the activity of AP-1 is regulated by JNK and extracellular signal-regulated kinase (ERK), of which the activation induces transcription of c-Fos and succeeding AP-1 activation. Through the activation of JNK and ERK signaling cascades, SARS induces an increase in the activity of AP-1, which regulates the transcription of many cytokine genes affected in SARS-CoV infection [80]. As Jun was targeted by wogonin, beta-sitosterol, kaempferol, and formononetin while Fos was targeted by quercetin, we suggest that the immunomodulatory effects of SZS may be mediated by Jun and Fos to protect against cytokine storm for COVID-19 treatment.

In addition to the potential therapeutic mechanism mentioned above, several targets of SZS, such as STAT3 and MAPK, were closely related to apoptosis (Figures 2 and 3). The transcriptional activity of STAT3 is influenced by viral infection and is relevant to the antiapoptotic activity of cells, and MAPK is reported to promote both cell death and survival [81, 82]. Moreover, Akt was the target with the highest degree among the 31 hub genes (Figure 2(b)), and the Akt signaling pathway is involved in cellular prosurvival signaling, which has been proven to be involved in the development of SARS-CoV infection [83]. The potential impact of SZS on cell death and survival during COVID-19 deserves further exploration.

### 3.4. SZS May Treat COVID-19 via Improving Respiratory Function

As indicated in the pathological results of COVID-19 [84], the diffuse alveolar damage with fibrin rich hyaline membranes and multinucleated giant cells, triggered by virus infection and inflammatory dysregulation, was the leading cause of diminished respiratory function like dyspnea and ARDS [84, 85]. Resulting from alveolar endothelial and epithelial barriers disruption, elevated vascular permeability ultimately leads to the dysfunction of the pulmonary alveolar-capillary barrier [86, 87]. After virus infection, the activation of the phosphoinositide 3-kinase (PI3K)/Akt/eNOS signaling pathway leads to an increase in histamine and NO, which increases microvascular permeability [88]. Sesamin is a lignan that exhibits antioxidative and anti-inflammatory effects by blocking the Toll-like receptor 4 signaling pathway and protecting human ECs in the lung [89]. Sesamin seems to alleviate histamine and NO-induced leakage by potently inhibiting Akt and p38 (MAPK) activities [90]. Moreover, we noticed that the histamine H1/2/3 receptors were simultaneously targeted by several compounds in SZS, including alloisoimperatorin, cnidilin, prangenidin, prangenin, and caribine, which probably hints at the potential role of SZS in regulating histamine-mediated microvascular permeability. Owing to the excessive fibronectin inhibited by the Serpine1 driven tissue-type plasminogen activators (PLAT), the elevated vascular permeability stimulates inflammatory cell migration and proliferation and recruits neutrophils to the lung, thus leading to severe lung injury [91]. Several compounds in SZS targeted Serpine1 and PLAT, such as quercetin, inflacoumarin A, 4,9-dimethoxy-1-vinyl-*β*-carboline, and kanzonol F (Table 1). Quercetin has been proven to increase the expression of PLAT while suppressing Serpine1 [92, 93]. Meanwhile, several active ingredients of SZS also targeted the Serpine1 and PLAT, including inflacoumarin A, 4,9-dimethoxy-1-vinyl-*β*-carboline, and kanzonol F (Table 1). In summary, SZS may play a protective role in maintaining the alveolar endothelial and epithelial barriers from disruption, relieving pulmonary capillary leakage, inhibiting the inflammatory environment of lung, and ultimately improving respiratory function.

There were some limitations in the present work. Since the latest update time of TCMSP and SymMap was 2014 and 2018, respectively, there may be some omissions of the components of SZS. Also, the dose-effect relationship of each component in SZS is not involved in these databases [94]. Such limitations remind us of treating the network pharmacological results more cautiously. At present, nevertheless, the network pharmacological approaches are still beneficial to research the mechanism of TCM in treating COVID-19.

## 4. Conclusion

In the present study, we investigated the underlying mechanism of the effects of SZS in treating the COVID-19 by utilizing the methods of integrating network pharmacology. The therapeutic effects potentially involve suppressing cytokine storms, protecting the pulmonary alveolar-capillary barrier, regulating the immune response, and mediating cell death and survival. Further experiments are needed to validate the specific molecular mechanisms of the effects of SZS on COVID-19.

## 5. Methods

### 5.1. Constructing Database of Candidate Compounds

The constituents of the compounds in SZS were retrieved from the online public databases Traditional Chinese Medicine Systems Pharmacology (TCMSP) (http://lsp.nwu.edu.cn/tcmsp.php) and SymMap (https://www.symmap.org/) [95–98]. Each candidate's druggability was analyzed according to its oral bioavailability (OB) and drug-likeness (DL). As an important indicator to objectively evaluate the intrinsic quality of drugs, OB is the degree and speed of the absorption of drugs into the circulatory system. The higher the OB of the compound is, the more likely the compound is to be applied clinically. DL refers to the sum of the pharmacokinetic and safety properties derived from the interactions of the physicochemical properties and structural factors, including solubility, permeability, and stability. The molecules with OB ≥ 30% and DL ≥ 0.18 were considered to exhibit relatively good pharmacokinetic properties and were identified as candidate compounds for analysis [99, 100]. All of the compound information was standardized according to “Canonical SMILES” based on the PubChem database (https://pubchem.ncbi.nlm.nih.gov/).

### 5.2. Predicting Targets of Active Compounds

The validated targets, which were confirmed by experiments, of the active ingredients of SZS were obtained from the TCMSP database [101]. Then, the potential target prediction of these active ingredients was performed by utilizing ChemMapper (http://www.lilab-ecust.cn/chemmapper/index.html), a web server for predicting potential drug targets based on the 3D structure similarity [102–105]. The similarity score is calculated from the molecular 3D similarity between the query and hit compounds and is scaled to [0, 2], while only five thresholds (0.8, 1.0, 1.2, 1.5, and 1.8, with 1.2 as the default value) can be set. All the compounds of which the score is below the threshold will not be exhibited. The closer the similarity score is to 2, the more likely a pharmacological link between the molecules is. In the present research, the similarity score threshold was set as the default value of 1.2 compared with drugs in the DrugBank database. Table S1 listed the targets of the active ingredients of SZS. As illustrated in the last column of Table S1, “validated” meant that the interactions of ingredients and targets were validated by the TCMSP database, while the others, which were shown in the form of prediction scores, were obtained from the ChemMapper. The prediction score is a standard score normalized to [0, 1]. The closer the prediction score is to 1, the higher confidence between the ingredient and the target is. Commonly, to screen the potential targets of the active compounds, the thresholds of prediction scores are set above 0, indicating good confidence [103–107]. To be specific, the prediction scores were outputted with three decimal places. If the score were still 0.000, which meant a very low level of confidence, the target would be identified as meaningless and removed. Moreover, duplicates of the validated and predicted targets were eliminated. In this way, the potential and rational targets of SZS could be taken into further analyses as far as possible, especially when the pathogenesis and therapeutic mechanism of COVID-19 are still not clear. All the targets obtained above were standardized according to their gene names and UniProt IDs by searching the UniProtKB (https://www.uniprot.org/) database with “Homo sapiens” as the species [108].

### 5.3. Constructing the Target Tissue Location Network

BioGPS is a database for querying and organizing gene annotation resources. It provides gene expression data for cells or tissues obtained by microarray analysis [32]. The target-organ location network was constructed with the use of the dataset: GeneAtlas U133 A, gcrma (http://biogps.org/#goto=welcome). First, the mRNA expression patterns of each target gene were determined in 84 different organ tissues. Second, the average values for each gene were analyzed. Third, the genes were located in the relevant organ tissues where the mRNA expression level was higher than the mean. Finally, a target-organ location network was constructed using Cytoscape 3.7.2 (https://cytoscape.org/), a well-known tool for network pharmacology research, to visualize the biological pathways and intermolecular interaction networks, among others. Furthermore, this supplied a basic set of features for data integration, analysis, and visualization for complex network analysis [109].

### 5.4. Constructing the Protein-Protein Interaction (PPI) Network

Since the chances of proteins performing their assigned functions individually are small, proteins involved in the biochemical process in the same cell tend to form macromolecular complexes through interactions to perform biological functions. Therefore, the exploration of protein interactions and their interaction networks are vital to understanding cellular organization, bioprocesses, and functions. In order to better understand protein interactions systematically, the associated targets were inputted into Search Tool for the Retrieval of Interacting Genes/Proteins (STRING, Version: 11.0) (https://string-db.org/) to obtain the relevant information on protein interaction, for which the genes were determined as nodes and the interactions as lines in a network [34]. STRING is an online database of functional protein association networks, providing associations between proteins according to experimentally determined data, literature mining, databases, and gene associations like neighborhood, fusions, co-occurrence, coexpression, or protein homology [110]. To ensure high interaction confidence, the minimum score was set to the highest confidence value of 0.9 to include the broad scope of protein interactions. Besides, disconnected proteins in the network were excluded. Finally, the protein interactions were determined according to exported statistics. The degree of a node indicates the number of routes to which the node is connected.

### 5.5. Gene Ontology (GO) Enrichment Analysis

Gene ontology (GO) is an international standardized gene functional classification system that offers a dynamically updated controlled vocabulary and strictly defined concepts to comprehensively describe the properties of genes and their products in any organism [35]. GO has three ontologies: molecular function (MF), cellular component (CC), and biological process (BP). The basic unit of GO is the GO term. Each GO term belongs to a type of ontology. GO enrichment analysis provides all GO terms that are significantly enriched in targets compared to the genome background and filters out the targets that correspond to biological functions. Firstly, all targets were mapped to GO terms in the gene ontology database (http://www.geneontology.org/), the gene numbers were calculated for every term, and significantly enriched GO terms in targets compared to the genome background were defined by a hypergeometric test. The calculated *p* value was then subjected to FDR correction. In this study, GO terms with an FDR ≤0.01, as the threshold, and the data were collected by ClueGO and CluePedia (Cytoscape plugins) [111–113]. Moreover, the targets were mapped for biological process (GO:0002376) and immune system process to explore the underlying impact on immune responses by utilizing Cytoscape equipped with the ClueGO and CluePedia plugins [38–40].

### 5.6. Pathway Enrichment Analysis

Genes usually interact with each other to play roles in certain biological functions. The pathway-based analysis helps to further understand gene biological functions. KEGG is the major public pathway-related database [114]. Pathway enrichment analysis identified significantly enriched metabolic pathways or signal transduction pathways involving targets compared with the whole genome background in the KEGG pathway database (http://www.genome.jp/kegg/). The calculated *p* value was subjected to FDR correction by taking FDR ≤0.01 as a threshold and the data were collected by the ClueGO and CluePedia plugins [115, 116]. Moreover, R software version 3.6.1 (http://www.r-project.org) with several R packages, including clusterProfiler, org.Hs.eg.db, enrichplot, and ggplot2, was applied to draw graphs during the GO and pathway enrichment analyses, such as bar plots, bubble diagrams, and a KEGG pathway map [117]. The R packages are available on Bioconductor (https://www.bioconductor.org/) [118].

### 5.7. Screening of Genes Related to SARS

Genes related to SARS were derived from MalaCards (https://www.malacards.org/pages/info), a database of human genes and genetic disorders. The proteins acquired from MalaCards were used as hub proteins and submitted to STRING and Human Protein Reference Database (HPRD) (http://www.hprd.org/) to determine the proteins interacting with these hub proteins. HPRD is a resource for experimentally derived information about the human proteome including protein-protein interactions, posttranslational modifications, and tissue expression [119, 120]. Meanwhile, we identified the gene expression data regarding SARS from the Gene Expression Omnibus (GEO) database (http://www.ncbi.nlm.nih.gov/geo/), an international public repository that archives and freely distributes high-throughput gene expression and other functional genomic datasets. The GEO dataset numbered GSE1739 is comprised of gene expression data obtained using the GPL201 Affymetrix Human HG-Focus Target Array from 14 samples, including 10 peripheral blood samples from adult patients with SARS and 4 controls. The workflow has been wrapped into the R packages “limma” and “edgeR” to identify the differentially expressed genes (DEGs) between the case and control samples [118]. In the current study, genes with an adjusted *p* value <0.05 and |LogFC|>1.0 were regarded as DEGs. Meanwhile, the R package “pheatmap” was used to draw the heatmap plot and volcano plot, with the up- and downregulated genes presented.

### 5.8. Construction and Analysis of Visualization Networks

In order to understand the complex relationship between active compounds and potential targets, the compound-target networks as well as the relationships among the compound-target-disease interactions were established with the software Cytoscape-v3.7.2 to reveal the molecular mechanisms. This network was composed of nodes and edges. Nodes represent molecules (compounds, targets, pathways, herbs, or diseases), and edges indicate intermolecular interactions, namely, the connections between nodes.

## Figures and Tables

**Table 1:** The compounds and targets of SZS.

Herb names	Number of components	Main components	Number of targets	Main targets
*Atractylodis Rhizoma (Cang Zhu * **苍术 **)	9	Wogonin	168	AKT1, JUN, VEGFA, MMP, TNF, IL6
		Stigmasterol 3-O-*β*-D-glucopyranoside_qt		
		*β*-sitosterol 3-O-glucoside_qt		
*Angelicae Dahuricae Radix (Bai Zhi * **白芷 **)	18	Alloisoimperatorin	228	AKT1, TLR4, JUN, FKBP, JAK
		Cnidilin		
		Prangenidin		
		Prangenin		
*Asari Radix Et Rhizoma (Xi Xin * **细辛 **)	6	Kaempferol	134	AKT1/2, JUN, MMP, PLAT, TNF, IL10
		Sesamin		
		Caribine		
		4,9-Dimethoxy-1-vinyl-*β*-carboline		
*Glycyrrhizae Radix Et Rhizoma (Gan Cao * **甘草 **)	71	Isotrifoliol	354	TLR4, JUN, VEGFA, PLAT, Serpine1, VCAM, IL10, STAT3, IL6, FKBP, FOS
		Inflacoumarin A		
		Kanzonol F		
		Quercetin		
		Formononetin		
*Notopterygii Rhizoma Et Radix (Qiang Huo * **羌活 **)	12	Cnidilin	157	MAPK8/10, AKT1, JUN, JAK
		*β*-sitosterol		
*Ligustici Rhizoma Et Radix (Gao Ben * **藁本 **)	1	Sitosterol	17	AR, ESR1, PGR
*Chuanxiong Rhizoma (Chuan Xiong * **川芎 **)	7	Perlolyrine	72	MAPK8, TLR7, NOS2, HCK, CA1
		Sitosterol		
		FA		

*Annotation*. The active compounds suffixed with “_qt”, called aglycone, are deglycosylated from the compounds connected with glycosyl groups, called glycosides, following the rule of the glycosidase hydrolysis reaction.

## Data Availability

The data used to support the findings of this study are available from the corresponding author upon request..

## Abbreviations

AJ: Adherens junctions
ARDS: Acute respiratory distress syndrome
BP: Biological process
CC: Cellular component
COVID-19: Coronavirus disease 2019
DEGs: Differentially expressed genes
DL: Drug-likeness
ECM: Extracellular matrix
GEO: Gene Expression Omnibus
GO: Gene ontology
HPRD: Human Protein Reference Database
JNK: c-Jun NH2-terminal kinases
MF: Molecular function
MMPs: Matrix metalloproteinases
OB: Oral bioavailability
PI3K: Phosphoinositide 3-kinase
PLAT: Plasminogen activators
PPI: Protein-protein interaction
ROS: Reactive oxygen species
SARS: Severe acute respiratory syndrome
SZS: Shen Zhu San
TCM: Traditional Chinese medicine
TCMSP: Traditional Chinese Medicine Systems Pharmacology
Th: T helper cell
TJ: Tight junctions
TNF-*α*: Tumor necrosis factor-*α*
USA: The United States of America
VCAM-1: Vascular cell adhesion molecule 1
WHO: World Health Organization.

## Supplementary Materials

Supplementary Materials Table S1: a database of component targets . Table S2: the statistics of GO functional enrichment of 627 targets. Table S3: the statistics of KEGG enrichment of 627 targets. Table S4: the statistics of GO functional enrichment of 40 cotargets in the immune system process. Table S5: the statistics of KEGG enrichment of 40 cotargets.
